# Gene expression and methylation profiles show the involvement of POMC in primary hyperparathyroidsm

**DOI:** 10.1186/s12967-022-03568-4

**Published:** 2022-08-16

**Authors:** Wen-Xuan Zhou, Shu Wang, Ting-Chao Wu, Ling-Chao Cheng, Yao Du, Wei Wu, Chen Lin, Xin-Ying Li, Zhong-Liang Hu

**Affiliations:** 1 Department of Pathology, School of Basic Medical Science, Central South University, Changsha, 410000 Hunan China; 2 Department of Breast Thyroid Surgery of Third Xiangya Hospital, Central South University, Changsha, 410000 Hunan China; 3 Department of Pathology, Xinjiang Medical University, Xinjiang, Urumqi, 830000 China; 4 Department of General Surgery, Xiangya Hospital, Central South University, Changsha, 410000 Hunan China; 5 Department of Pathology, Xiangya Hospital, Central South University, Changsha, 410000 Hunan China

**Keywords:** Primary hyperparathyroidism, Transcriptome sequencing, DNA methylation sequencing, POMC

## Abstract

Primary hyperparathyroidism (PHPT) is mainly caused by parathyroid adenoma, which produces excess parathyroid hormones. Its pathogenic mechanisms have not yet been fully understood. To investigate the mechanism in the pathogenesis of PHPT, the transcriptome and genome-wide DNA methylation profiles of parathyroid adenoma were analyzed. The candidate genes that may be involved in the PHPT were verified via qRT-PCR, immunohistochemistry, western blot, and methylation-specific PCR. A total of 1650 differentially expressed genes and 2373 differentially methylated regions were identified. After the integration of its transcriptome and DNA methylation data, IL6, SYP, GNA01, and pro-opiomelanocortin (POMC) were the candidate genes that demonstrated a similar pattern between their mRNA expression and DNA methylation status. Of the 4 candidate genes, POMC, a pro-peptide which is processed to a range of bioactive peptide products like ACTH, was further confirmed to be expressed at low levels at both the mRNA and protein levels, which may be due to POMC promoter hypermethylation. Hypermethylation of the POMC promoter may contribute to its low expression, which may be involved in the pathogenesis of PHPT.

**Supplementary Information:**

The online version contains supplementary material available at 10.1186/s12967-022-03568-4.

## Background

Primary hyperparathyroidism (PHPT), caused by hypersecretion of parathyroid hormone (PTH) in one to four parathyroid glands, is a common endocrine disorder that is characterized by chronic elevation of serum concentrations of calcium [1, 2]. PHPT can occur at any age, but half of them are present in postmenopausal women [3]. Population-based data from the United States show an annual incidence of 66 cases per 100,000 population in women and from 13 to 36 per 100,000 population in men [4]. Parathyroidectomy remains the definitive management for PHPT [5]. The pathological phenotype of PHPT is diverse, with solitary parathyroid adenomas seen in approximately 80% of cases. Double adenoma together with hyperplasia of all glands is present in approximately 15% of cases [6], while less than 1% of cases are caused by parathyroid cancer. The pathogenesis of parathyroid tumors is a multistage and complex process that is generally triggered by single or multiple mutations or deletions involved in one or more genes [7, 8]. Moreover, epigenetic modifications, such as DNA methylation, may also play a key role in the development of parathyroid tumors [9].

However, the pathogenesis of PHPT is not yet fully understood. It is imperative to elucidate the crucial molecular markers and pathways underlying the initiation and development of PHPT, which may help the development of targeted therapies [10]. Next-generation sequencing (NGS) technology has been widely applied to transcriptome and epigenetic analysis [11]. To investigate the pathogenesis of PHPT, we collected parathyroid tissues, including 2 normal tissues and 6 parathyroid adenomas, for RNA-seq and genome-wide DNA methylation sequencing.

## Materials and methods

### Tissue specimens

Fresh parathyroid adenoma tissues (n = 12) were harvested from 12 patients with PHPT, while normal parathyroid tissue (n = 10) was obtained as normal parathyroid gland inadvertently removed in patients subjected to thyroid cancer surgery (Table 1). The inclusion criteria of PHPT cases were as follows: serum PTH levels ≥ 70 pg/ml with hypercalcemia or blood calcium at the upper limit of normal and surgically as well as pathologically confirmed parathyroid adenoma.

**Table 1:** Sample ID used for analysis

Source	Samples	Quantity	No
Fresh tissues	Normal	10	N01,N02……N10
	PHPT	12	P01,P02…….P12
paraffin-embedded tissues	Normal	11	N11,N12……N21
	PHPT	31	P13,P14……P43

For immunohistochemistry and methylation-specific PCR, formalin-fixed and paraffin-embedded tissues from a total of 42 patients were obtained from the Department of Pathology, Xiangya Hospital (Table 1, details are shown on Additional file 1: Table S1). Informed consent and approval of the Xiangya Hospital ethics committee (Approval Number: 20180223-174; data: 02-23-2018) were archived.

### RNA-Sequencing and data processing

Total RNA was obtained from the selected tissue samples using the RNeasy Protect Mini Kit (Qiagen, details of all the reagents can be found on Additional file 2: Table S2) following the manufacturer's instructions. RNA was delivered to Beijing Genomics Institute (BGI, China) for single-end RNA-seq assay and profiling by a BGISEQ-500 sequencer. The depth of sequencing in our experiment was 24 M reads per sample. Quantitative gene expression analysis and differential analysis were performed using the RSEM tool and Noiseq method. We screen differentially expressed genes (DEGs) according to the following default criteria: fold change ≥ 2 and diverge probability ≥ 0.8. GO functional and pathway enrichment analyses for differentially expressed the AimGO2 database [12] (http://amigo.geneontology.org/amigo) and KEGG pathway database [13] (https://www.genome.jp/) by Phyper based on Hypergeometric test. The significant levels of terms and pathways were corrected by Q value with a rigorous threshold (Q value ≤ 0.05) by Bonferroni [14].

### PPI network construction and analysis

Protein–protein interaction data were obtained from STRING (https://string-db.org/). |Log2FC|≥ 2 was set as the restrictive condition to screen DEGs. In our study, only interactions with weights above the cutoff value of 0.4 were selected for the newly constructed PPI network.

Cytoscape was used to construct the PPI network. CytoHubba [15], as a Cytoscape plugin, was applied to calculate topological features of the PPI network, and another Cytoscape plugin, MCODE [16], was used to identify subnetworks. The genes obtained in either CytoHubba or MCODE are combined together and are the key genes of DEGs.

### Whole-Genome Bisulfite Sequencing (WGBS) and data analysis

Total genomic DNA (gDNA) was isolated using a modified cetyltrimethylammonium bromide (CTAB) method. Purified DNA was delivered to BGI for whole genome bisulfite sequencing (WGBS). The fragmented DNA was selected by an Agencourt AMPure XP-Medium kit. The selected fragments were subjected to end repair, 3ʹ-adenylation, adapter ligation, bisulfite treatment, PCR amplification, and library construction. Sequencing was performed using the BGISEQ-500 platform. The filtered reads were mapped to the human reference genome using BSMAP software.

The methylation level for each cytosine (CG, CHH or CHG) was calculated. Differentially methylated regions (DMRs) containing at least five methylated cytosine sites were identified. CIRCOS was used to compare the differences in the methylation levels of DMRs between the samples. Finally, the GO (http://amigo.geneontology.org/amigo) and KEGG (https://www.kegg.jp/) enrichment of DMRs-related genes was performed using phyper to calculate p-value, cooperate with the false discovery rate (FDR) by Bonferroni [14].

All the screened key genes were compared with the methylation sequencing results. The overlapping genes in key genes and DMRs were defined as the hub genes.

### Real-time quantitative polymerase chain reaction (qRT-PCR)

Total RNA of parathyroid tissues was extracted with an RNeasy Protect Mini Kit (Qiagen) to synthesize cDNA using the All-in-One^™^ First-Strand cDNA Synthesis Kit (GeneCopoeia). RT-PCR was performed on a Fast 7500 Real-Time PCR system (ABI, USA). The PCR conditions were as follows: denaturation at 95 ℃ for 10 s and annealing and elongation at 60 ℃ for 30 s. Relative expression was analyzed using the DDCt method. The primer are designed by the software on the Sangon Biotech (https://www.sangon.com/). Sequences are listed on Table 2.

**Table 2:** qRT-PCR primers for the POMC gene

Primer Name	Primer sequences	Product size
POMC-F	CTCCCGAGACAGAGCCTCA	168 bp
POMC-R	ACTCCAGCAGGTTGCTTTCC	
GAPDH-F	CCATGGGTGGAATCATATTGGA	139 bp
GAPDH-R	TCAACGGATTTGGTCGTATTGG	

### Western blot

Protein was extracted in cell lysis buffer containing 1% protease inhibitor cocktail and then quantified by an enhanced BCA protein assay kit (Beyotime, China). Equal amounts of proteins were separated by 12% sodium dodecyl sulfate-polyacrylamide gel electrophoresis (SDS-PAGE). Membranes were blocked with PBST buffer containing 5% nonfat skim milk. Immunostaining was performed overnight at 4 °C using primary antibodies [POMC (1:2500 dilution; Abcam); Affinity (1:2000 dilution, Proteintech)]. After incubation with horseradish peroxidase–conjugated secondary antibodies, the proteins were visualized using enhanced chemiluminescence (ECL, Bio-Rad, USA) following the manufacturer’s instructions. ImageJ software was used to quantify protein band intensity.

### Immunohistochemistry (IHC)

Formalin-fixed, paraffin-embedded parathyroid tissue sections were used for IHC staining according to the method described in some previous studies [17, 18]. Heat-induced epitope retrieval was performed with EDTA buffer (pH 9.0). In brief, the sections were first incubated with primary antibodies against POMC (1:500 dilution; Abcam), SYP (1:200 dilution; Cell Signaling), IL6 (1:500 dilution, Proteintech), or GNAO1 (1:500 dilution, Proteintech), followed by secondary antibody and diaminobenzidine (DAB, Vector Laboratories, Burlingame, CA) staining. The nuclei were counterstained with Meyer’s hematoxylin. Immunohistochemistry slides were scanned and analyzed via the digital pathology system and then quantified with the image analysis tool ImageJ, which reports the average optical density (AOD) of each image.

### Methylation-specific-solymerase chain reaction (MS-PCR)

In all, 16 samples were analyzed by MS-PCR. Total DNA from paraffin-embedded tissues of parathyroid glands was extracted using the QIAamp DNA paraffin-embedded Tissue Kit (Qiagen), and genomic DNA was treated with bisulfite using the EZ DNA Methylation-Gold Kits (Zymo Research). All unmethylated cytosine residues in DNA were converted into uracil, with no influence on methylated cytosine. Specific primers for both methylated and unmethylated DNA sequences were used for the POMC promoter (Table 3). Subsequently, promoter methylation of the POMC gene was detected by MS-PCR. The amplification products were detected by 2% agarose gel electrophoresis, and PCR results were analyzed on a Gel Imager.

**Table 3:** Primers for the POMC gene

Primer name	Primer sequences	Product size
POMC-M-F	TAGTTTTTAAATAATGGGGAAATCG	141 bp
POMC-M-R	AACAACCTCTAAAATCGTTAAAACG	
POMC-U-F	ATAGTTTTTAAATAATGGGGAAATTG	140 bp
POMC-U-R	CAACCTCTAAAATCATTAAAACAAA	

### Statistical analysis

Data from qPCR, western blot, and IHC were analyzed by Student’s t-test. All differences with P-value < 0.05 were considered statistically significant.

## Results

### Differentially expressed genes between normal parathyroid and parathyroid adenoma

**Fig. 1:**
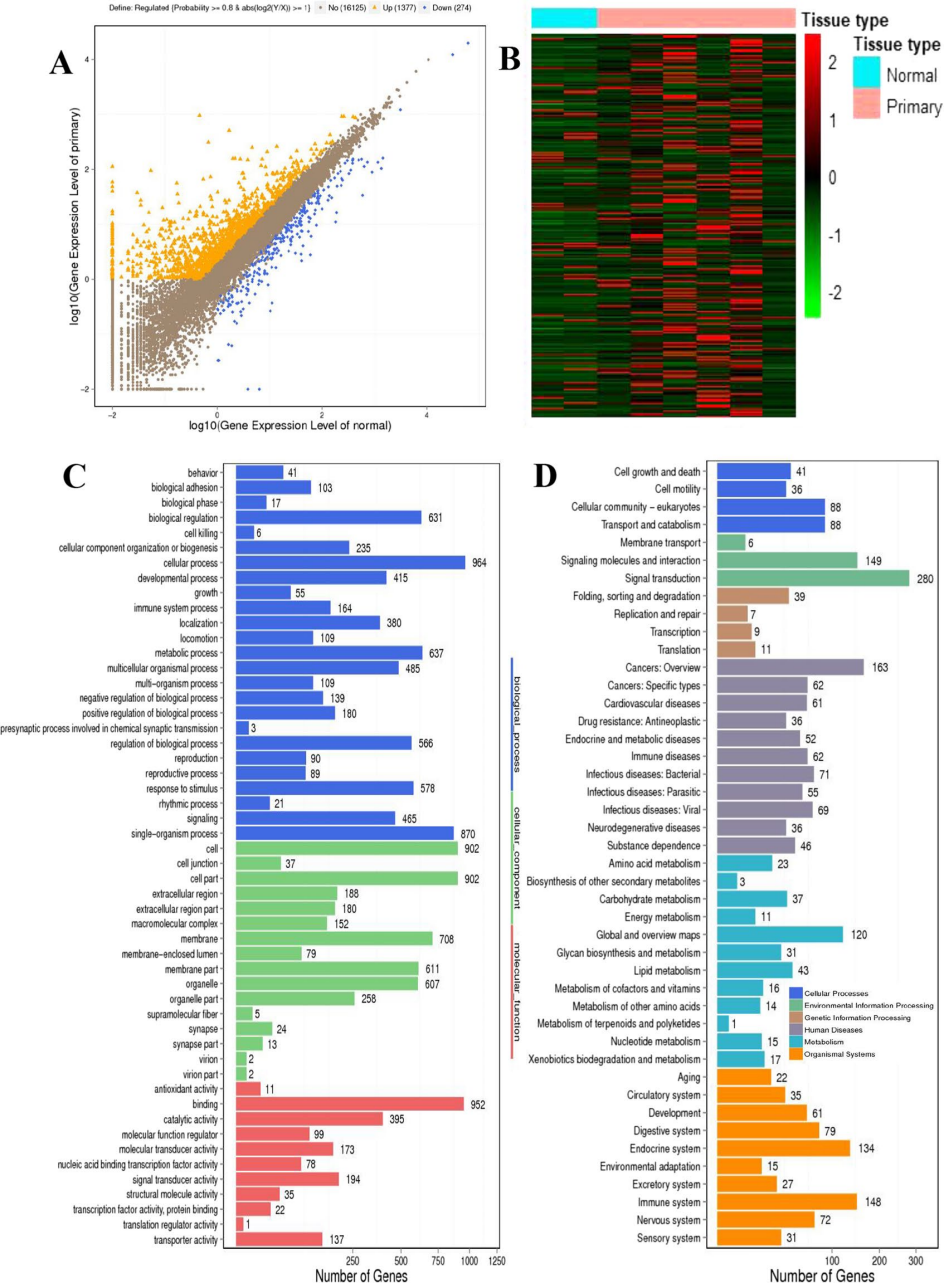

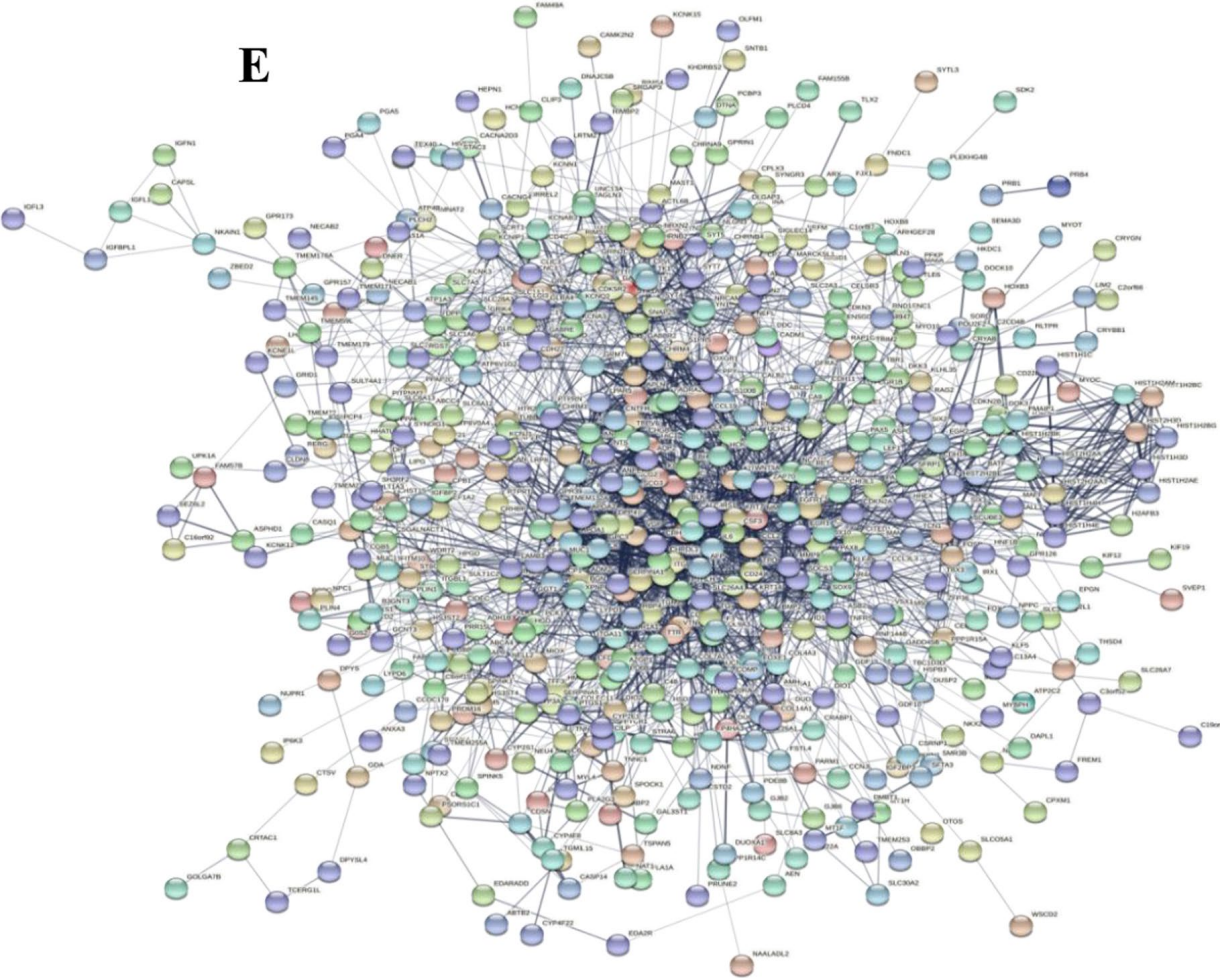
Differentially expressed genes between normal parathyroid and parathyroid adenomas. **A** Scatter plots of DEGs. Orange indicates upregulation, blue downregulation, and brown indicates no change. Two normal parathyroid tissues (N01-N02) and 6 parathyroid adenomas (P01-P06) were used for transcriptome sequencing. **B** Heat map of 676 DEGs with |log2FC|≥ 2. Different types of DEGs are represented by different colors. Red indicates upregulation, and green downregulation. **C** GO analysis of DEGs. **D** KEGG enrichment analysis of DEGs. **E** PPI network of 583 selected DEGs

A total of 1650 DEGs were identified, including 1376 upregulated genes and 274 downregulated genes (Fig. 1A), and 676 DEGs with |log2FC|≥ 2 were used to make a heat map (Fig. 1B). A total of 1650 DEGs were used for GO enrichment analysis covering biological processes, cellular components, and molecular functions. As shown in Fig. 1C, many biological processes, mainly cellular processes, single-organism processes, and metabolic processes, were apparent in the pathogenesis of PHPT. The differential cellular components were distributed on the cell, cell part, and cell membrane. Molecular functions focused on binding, catalytic activity and signal transducer activity. The top 3 differentially enriched KEGG pathways (Fig. 1D) were signal transduction, cancers, and immune.

A total of 676 DEGs with |log2FC|≥ 2 were used to construct the PPI network. After filtering, 93 of them were excluded due to low interactions with other genes. Finally, 583 nodes (DEGs) and 2762 edges were revealed in the PPI network (Fig. 1E). To obtain the key genes in the PPI network, two apps in Cytoscape software were used. First, by applying 12 topological algorithms in CytoHubba, 17 key genes were extracted, consisting of AFP, ALB, APOA1, HIST1H2AE, HIST1H2AM, KIT, IL6, CCL2, FOS, NTRK2, MMP9, CAM1, POMC, SNAP25, SYP, BMP2, and GNAO1. Then, 6 seed genes were obtained using MCODE, which calculated the regions in the PPI network, including TMEM132A, IER2, P4HA3, CADPS, SYT5, and CIDEC. Therefore, 23 key genes were identified after the analysis of Cytoscape and MCODE.

### Differential DNA methylation between normal parathyroid and parathyroid adenoma

**Fig. 2:**
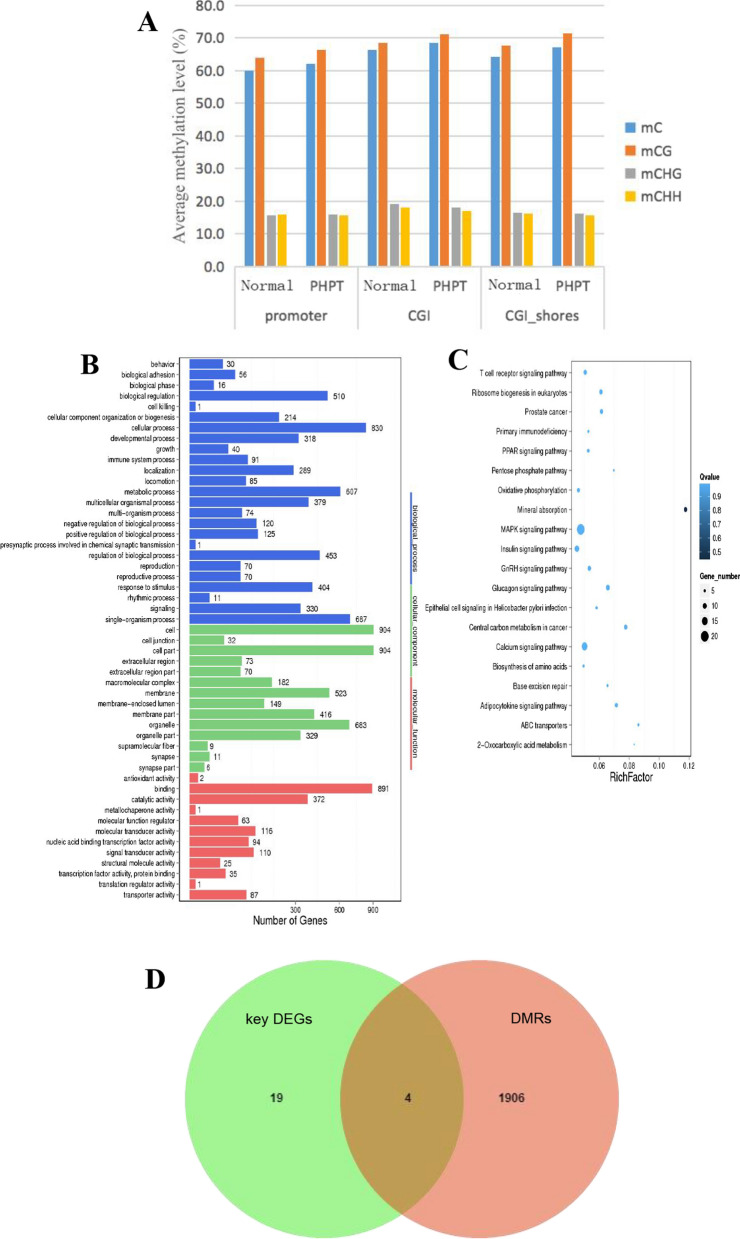
Differentially methylated regions between normal parathyroid and parathyroid adenomas. **A** Average methylation levels of different genomic regions. The X axis indicates the average methylation levels of three different genomic regions, including promoters (promoters), CpG islands (CGIs), and 2 K regions downstream of CpG islands (CGI_shores), in normal parathyroid tissue (normal) and PHPT tissue (PHPT). Different colors represent different types of methylated C sites. Two normal parathyroid tissues (N03-N04) and 6 parathyroid adenomas (P01-P06) were used for WGBS. **B** GO analysis of DMRs. **C** KEGG enrichment analysis of DMRs. **D** Venn diagram analysis of 23 key DEGs and all the DMRs

DNA methylation is a form of DNA modification that can control gene expression and alter genetic expression without altering the DNA sequence. To determine whether DEGs were associated with methylation of their promoter region, tissues from the same patients with parathyroid adenomas used for RNA sequencing were used for DNA methylation sequencing. The average methylation level of cytosine in different genomic regions was analyzed. In each genomic region of both normal parathyroid and parathyroid adenoma, the mC frequency was highest at CG sites, ranging from 63 to 71%, and was lower at CHG and CHH sites, with 15–19% and 15–18%, respectively. Among the different genomic regions, the CpG island (CGI) had the highest methylation level, suggesting that this region may be an epigenetic regulatory region that alters gene expression. As shown in Fig. 2A, the methylation level of parathyroid adenoma tissue was significantly higher than that of normal parathyroid tissue, both in the CG background and in the different genetic regions.

Compared to the methylation profiles of normal parathyroid tissue, a total of 2373 differentially methylated regions (DMRs) were identified in parathyroid adenoma. Among these DMRs, hypomethylated DMRs are much more frequent than hypermethylated DMRs, suggesting that hypomethylation was observed more frequently than in parathyroid adenoma tissue compared to normal parathyroid tissue. GO analysis indicated that the differential components were distributed in cells, cell parts and binding (Fig. 2B). KEGG analysis indicated that DMRs were mainly enriched in the mineral absorption pathway (Fig. 2C).

### Integrated analysis of RNA-seq and DNA methylation data

To investigate the similar results between RNA-seq data and DNA methylation data, we compared 23 key genes expression in PPI network with their DNA methylation results (Fig. 2D), four genes showed similar results between RNA-seq data and DNA methylation data, with high mRNA and low promoter methylation, or low mRNA and high promoter methylation, including IL6, POMC, SYP, and GNA01. Both the expression of IL6 and POMC mRNA was decreased with hypermethylation at their promoter regions, while both SYP and GNA01 showed hypomethylated promoters and high mRNA levels.

### Low expression and hypermethylation of POMC in parathyroid adenoma.

**Fig. 3:**
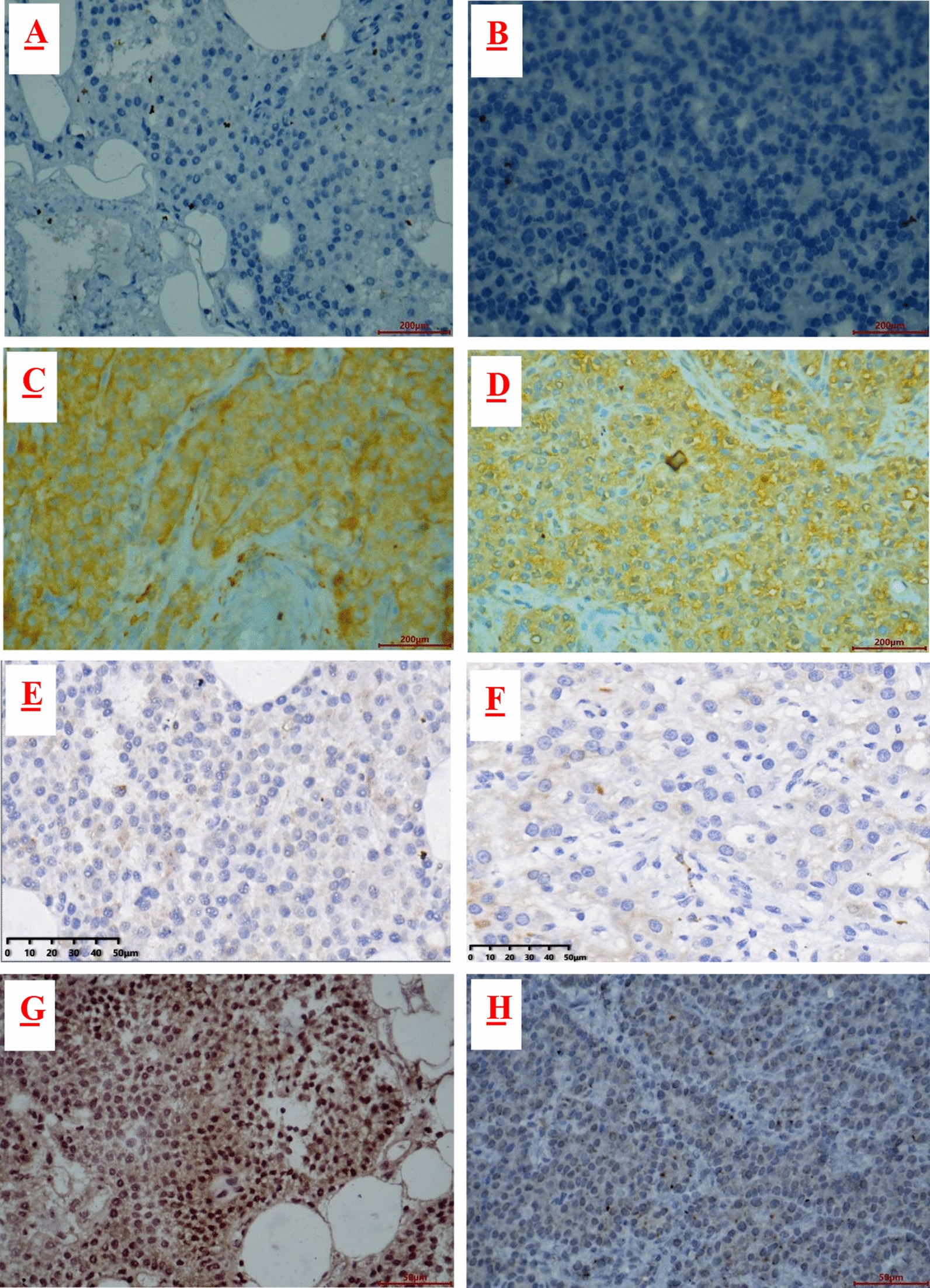

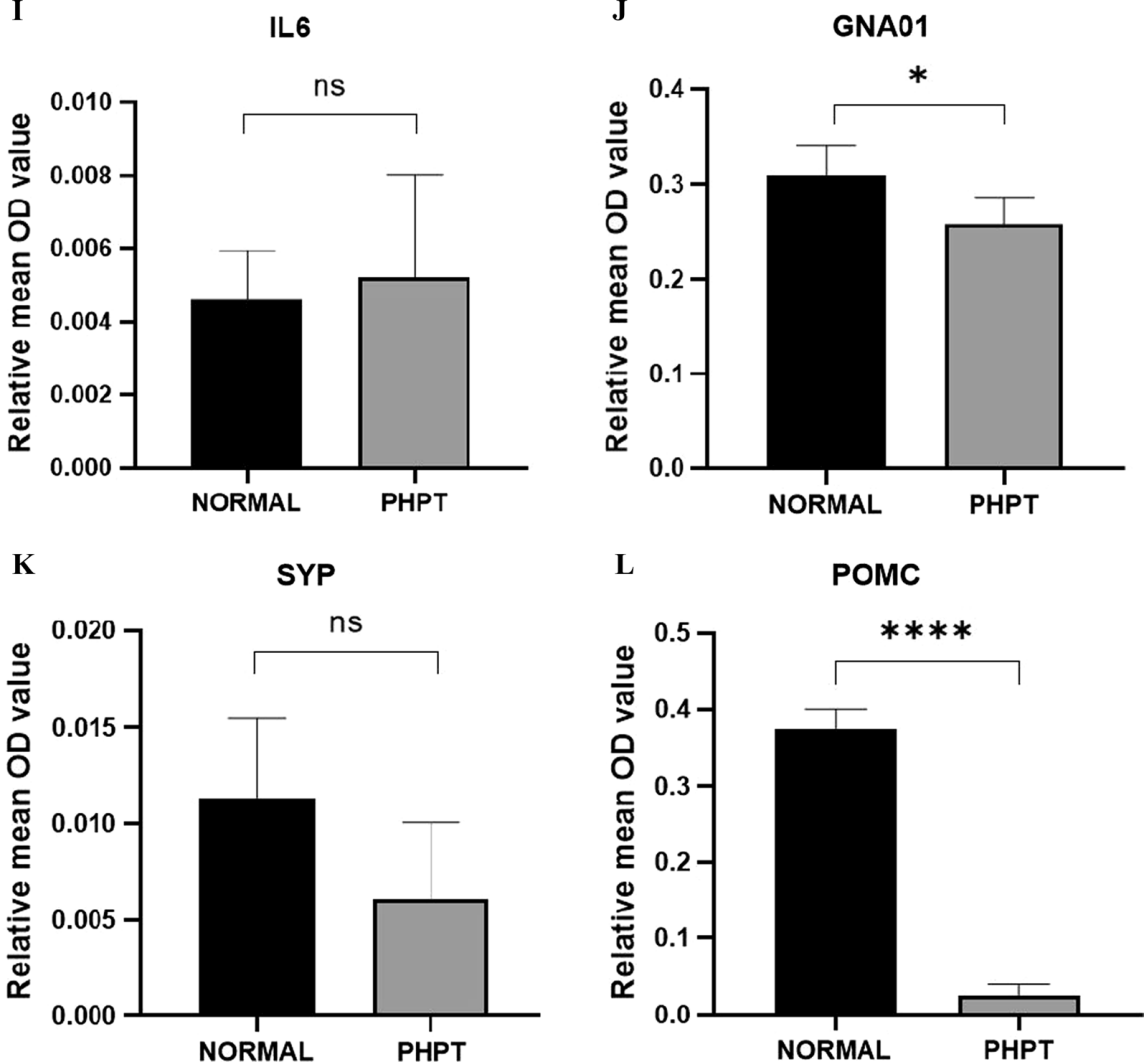
Protein expression of four hub genes by immunohistochemistry. Protein expression levels of IL-6, GNAO1, SYP and POMC in normal parathyroid tissue (**A**, **C**, **E**, **G**) and PHPT parathyroid tissue (**B**, **D**, **F**, **H**). Five normal parathyroid tissues (N11–N15) and 10 parathyroid adenomas (P13–P22) were chosen for this experiment. The quantification were shown on **I**–**L**. ns: no significant. *P < 0.05. ****P < 0.0001

The expression of IL6, POMC, SYP, and GNA01 was detected via immunohistochemistry (Fig. 3). Compared to normal parathyroid tissues, the expression of IL6, GNA01, and SYP showed no difference in parathyroid adenomas. However, the expression of POMC in parathyroid adenomas was markedly lower than that in normal parathyroid tissue. Therefore, POMC was selected for further experiment.

**Fig. 4:**
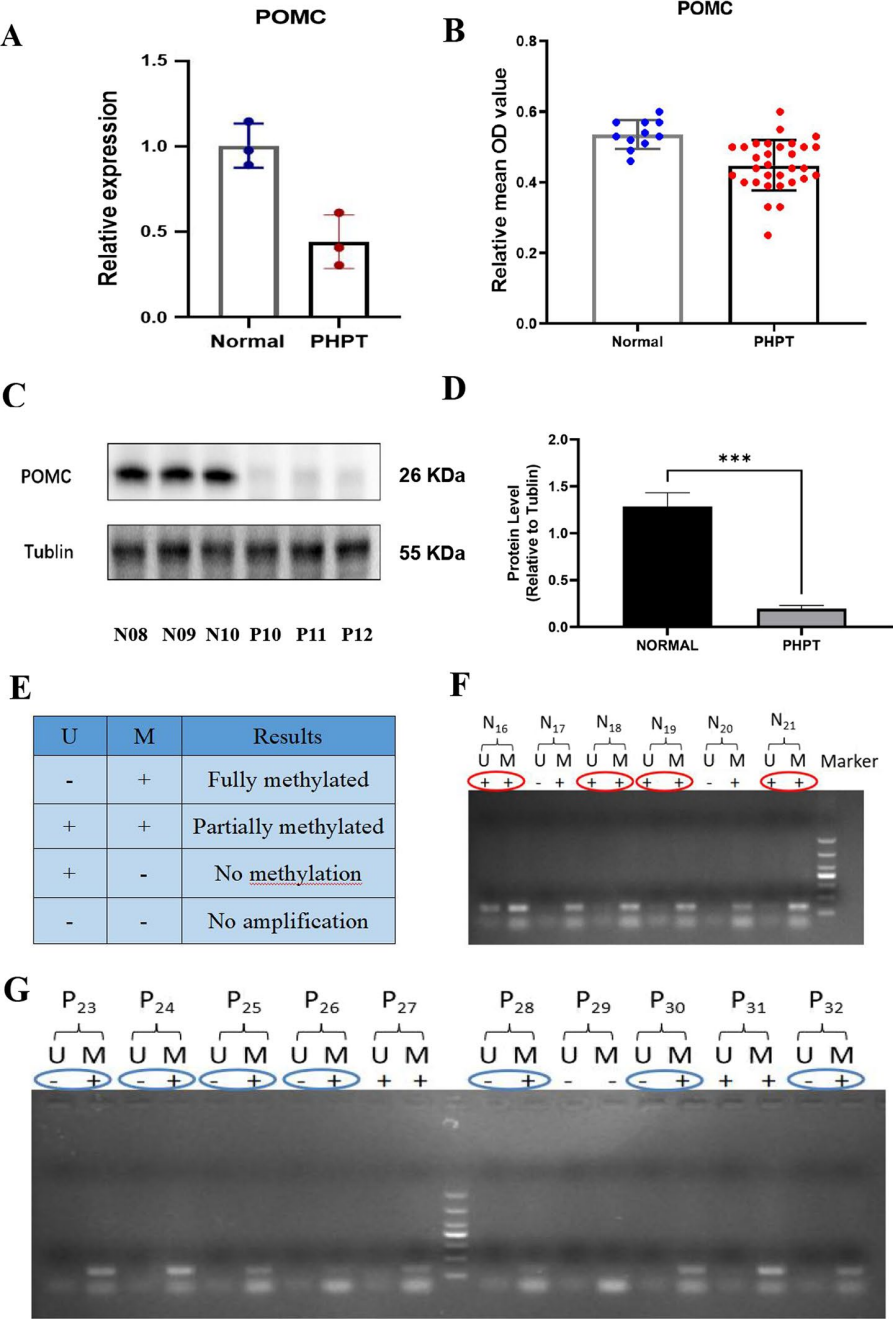

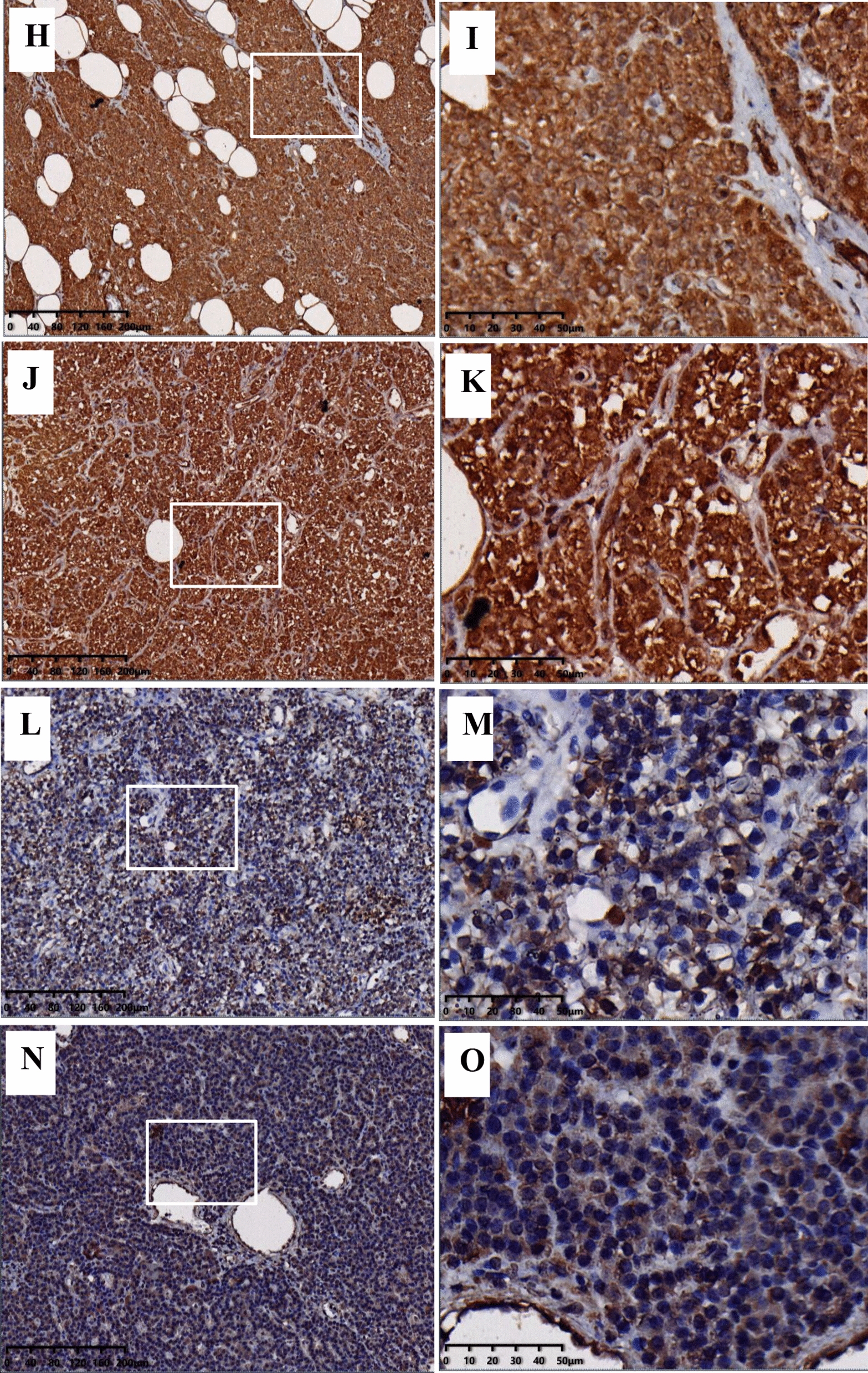

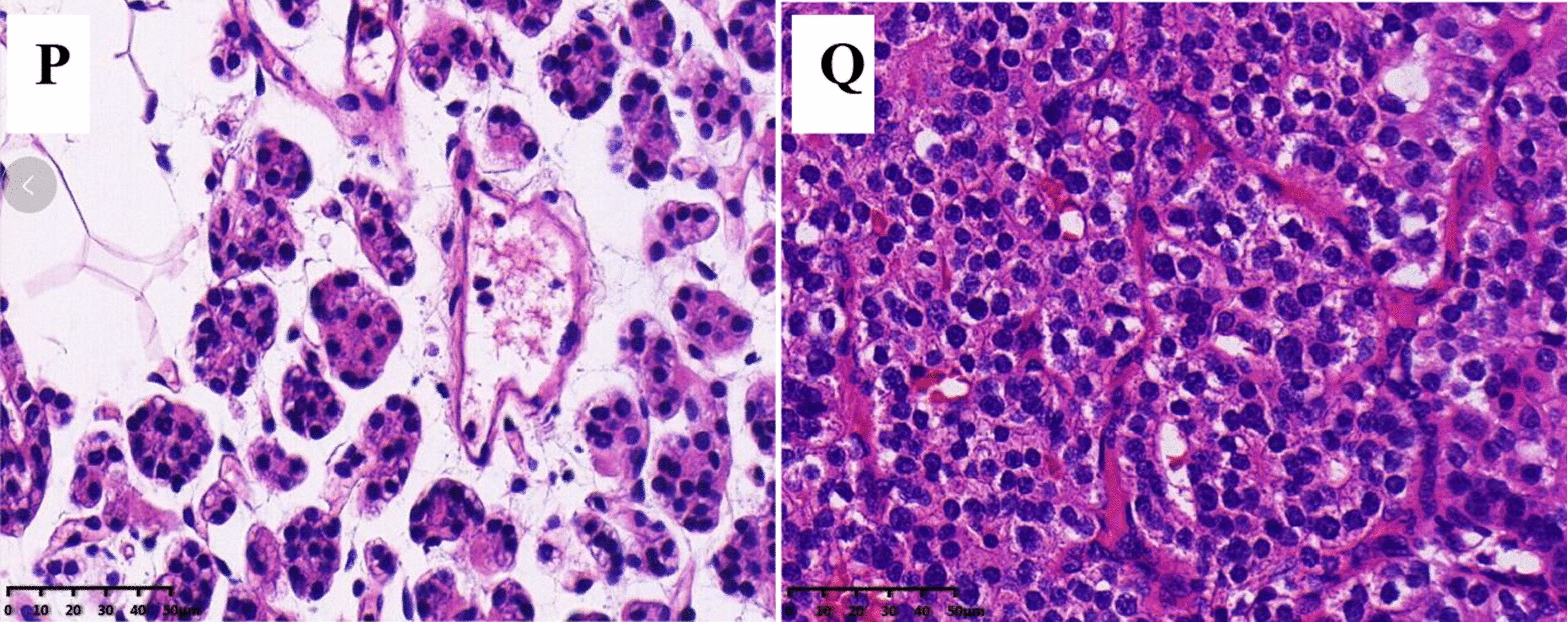
Expression of POMC in the PHPT and normal cohorts. **A** POMC mRNA expression between 3 normal parathyroid samples (N05–N07) and 3 parathyroid adenoma samples (P07–P09) by qRT-PCR. *P* = 0.0086. **B** POMC protein expression in the normal and PHPT cohorts by immunohistochemistry. Paraffin-embedded tissues from 11 normal parathyroid samples (N11–N21) and 31 parathyroid adenomas (P13–P43) were analyzed. *P* = 0.0005. **C** POMC protein expression in normal parathyroid tissue (N08–N10) and PHPT parathyroid tissue (P10–P12) by western blot and was shown as histogram (**D**). *P* = 0.0002. **E** The methylation levels of the POMC promoter region in normal parathyroid tissue **(F)** and parathyroid adenoma **(G)**. There were 16 samples for the experiment, including 6 normal parathyroid tissues (N16–N21) and 10 PHPT parathyroid tissues (P23–P32). Each experiment was done in triplicate. M represents the amplification products with the methylated primers. U indicates the amplification products with the unmethylated primers. POMC expression in normal parathyroid glands (**H, I, J, K**) and parathyroid adenoma (**L, M, N, O**) via immunohistochemistry. The right series of microphotographs (**I, K, M, O**) were taken from the parts shown in the white boxes in the left microphotographs (**H, J, L, N**). Hematoxylin and eosin staining of normal parathyroid glands (**P**) and parathyroid adenoma (**Q**). Scale bar: 50 um.

The mRNA expression of POMC in parathyroid adenoma was validated via qRT-PCR. As shown in Fig. 4A, POMC expression was significantly reduced compared with that in normal parathyroid tissues. The expression of POMC protein was analyzed in normal parathyroid and parathyroid adenoma FFPE samples via immunohistochemistry. Compared to normal parathyroid adenomas, the expression of POMC protein in parathyroid adenomas was significantly decreased (Fig. 4B). Western blot showed similar results (Fig. 4C, D). In addition, POMC protein in parathyroid adenomas was mainly expressed in the cytoplasm, while in normal parathyroid samples, it was also expressed in the nucleus (Fig. 4H–Q). To further confirm the POMC methylation status, MSP, a rapid sensitive and specific method for detecting the methylation status of any set of CpG sites on CpG islands, was performed. Our results showed that most of the normal group (4/6) demonstrated partial methylation, while most of the parathyroid adenoma (7/10) was fully methylated (Fig. 4E–G).

## Discussion

Primary hyperparathyroidism (PHPT) is common in adults [19]. Multiple complications will arise because of the disruption of calcium homeostasis. However, currently, many patients present with nonspecific symptoms and may even be asymptomatic. PHPT can be diagnosed early by measuring calcium and parathyroid hormone levels [20]. Previous studies have suggested that its proliferative changes have several different mechanisms: epigenetic alterations, oncogene mutations and oncogene activation regulation [21, 22]. In our study, RNA-seq and DNA methylation sequencing were performed on parathyroid adenoma tissues, and then DEGs and DMRs were identified. After constructing a PPI network with 583 screened DEGs and calculating with Cytoscape software, 23 key genes were identified. After the intersection between DEGs and DMRs and further validation, we found that the expression of POMC in PHPT tissue was significantly lower than that in normal parathyroid tissue.

Pro-adrenocorticotropin (POMC) produces a number of active peptides, such as adrenocorticotropic hormone (ACTH) [23], to regulate the HPA (hypothalamic–pituitary–adrenal) axis [24]. It has been shown that patients with PHPT do have abnormalities in the HPA axis [25] caused by high concentrations of PTH and calcium, which further stimulate adrenal cAMP to promote cortisol and aldosterone secretion. This role of PTH is attributed to its structural similarity of 15–25 amino acids to 1–11 amino acids of ACTH and the indirect effect of hypercalcemia [26]. PHPT is characterized by hypercalcemia as well as inappropriately elevated PTH, both of which may alter the functional status of the HPA axis. The association between PHPT and the HPA axis indicates that POMC may influence PHPT through the HPA axis.

Among epigenetic modifications, the most characteristic is DNA methylation. The vast majority of DNA methylation in the human genome occurs in promoter CpG islands, and the hypermethylation of promoters is mainly associated with transcriptional repression [27]. In our study, compared to normal parathyroid tissue, parathyroid adenoma showed hypermethylation of the CpG island in POMC.

Several studies have suggested that the expression of the POMC gene is regulated by hypermethylation of the POMC promoter region [28–30]. Our experiments also indicated that hypermethylation of the POMC promoter region contributes to its low expression in PHPT.

To our knowledge, this is the first study to investigate the relationships between POMC and PHPT. However, in our experiments, the samples were quite limited. Cell biology experiment and animal models of PHPT are necessary to explore the role of POMC in the pathogenesis of PHPT.

## Conclusions

Hypermethylation of the POMC promoter may contribute to its low expression, which may be involved in the pathogenesis of PHPT.

## Supplementary Information

**Additional file 1: Table S1**: Details of 43 patients’ samples.

**Additional file 2: Table S2**: Key Resources table.

## Data Availability

The datasets supporting the conclusions of this article are available in the Sequence Read Archive (SRA) repository under accession code PRJNA754691 and PRJNA752238.
