# Supplementary material for: Gene expression and methylation profiles show the involvement of POMC in primary hyperparathyroidsm
Source: J Transl Med. 2022 Aug 16;20:368. doi: 10.1186/s12967-022-03568-4 (PMC9382844; doi:10.1186/s12967-022-03568-4)
Supplement: Supplementary file 1 — Additional file 1: Table S1: Details of 43 patients’ samples. [file 12967_2022_3568_MOESM1_ESM.pdf]

Table S1 Details of 43 patients' samples

	Sample ID	Gender	Age (years old)	Menopause or not
Fresh tissues	N01	Female	60	yes
	N02	Male	45	
	N03	Male	35	
	N04	Female	32	no
	N05	Female	39	no
	N06	Female	34	no
	N07	Male	40	
	N08	Female	39	no
	N09	Female	38	no
	N10	Male	54	
	P01	Female	44	no
	P02	Female	27	no
	P03	Male	43	
	P04	Female	47	yes
	P05	Male	67	
	P06	Male	39	
	P07	Male	58	
	P08	Female	53	yes
	P09	Female	42	no
	P10	Female	52	yes
	P11	Male	52	
	P12	Female	11	no
paraffin-embedded tissues	N11	Male	38	
	N12	Female	30	no
	N13	Female	53	yes
	N14	Male	26	
	N15	Male	39	
	N16	Male	43	
	N17	Male	25	
	N18	Female	53	yes
	N19	Male	18	
	N20	Female	28	no
	N21	Female	37	no
	P13	Female	73	yes
	P14	Female	63	yes
	P15	Female	35	no
	P16	Female	22	no
	P17	Female	42	no
	P18	Male	25	
	P19	Female	50	yes
	P20	Female	61	yes
	P21	Female	38	no

	Sample ID	Gender	age	Menopause or not
paraffin-embedded tissues	P22	Female	58	yes
	P23	Female	33	no
	P24	Female	13	no
	P25	Male	47	
	P26	Male	24	
	P27	Male	37	
	P28	Male	47	
	P29	Male	73	
	P30	Male	53	
	P31	Female	50	no
	P32	Female	46	no
	P33	Male	41	
	P34	Male	65	
	P35	Female	69	yes
	P36	Male	67	
	P37	Male	18	
	P38	Female	44	no
	P39	Female	46	yes
	P40	Male	18	
	P41	Female	54	yes
	P42	Female	65	yes
	P43	Female	54	yes
