# Supplementary material for: Gene expression and methylation profiles show the involvement of POMC in primary hyperparathyroidsm
Source: J Transl Med. 2022 Aug 16;20:368. doi: 10.1186/s12967-022-03568-4 (PMC9382844; doi:10.1186/s12967-022-03568-4)
Supplement: Supplementary file 2 — Additional file 2: Table S2: Key Resources table. [file 12967_2022_3568_MOESM2_ESM.pdf]

Table S2 Key Resources table

Reagent (alphabetical order)	Supplier	Cat.No.
All-in-One™ First-Strand cDNA Synthesis Kit	GeneCopoei	AORT-0020
All-in-One™qPCR Mix	GeneCopoei	AOPR-0200
Anti-POMC antibody [EPR17571]	Abcam	ab210605
Anti-POMC antibody	Abcam	ab32893
Clarity Western ECL Substrate	Bio-Rad	1705060
DAB solution	Vector Laboratories	SK-4105
Enhanced BCA Protein Assay Kit	Beyotime	P0010
EZ DNA Methylation-Gold Kits	Zymo Research	D5005
Goat Anti-Rabbit IgG (H+L) HRP	affinity	S0001
GNAO1 Polyclonal antibody	Proteintech	12635-1-AP
IL-6 Monoclonal antibody	Proteintech	66146-1-Ig
Protease Inhibitor Cocktail （100x）	CWBIO	CW2200
QIAamp DNA FFPE Tissue Kit	QIAGEN	56404
RIPA buffer(high)	Solarbio	R0010
RNeasy Protect Mini Kit (50)	QIAGEN	74124
Synaptophysin (D8F6H) XP® Rabbit mAb	Cell Signaling	36406
Tris-EDTA Antigen Repair Solution（10×） pH 9.0	Solarbio	C1038
Tubulin beta Antibody	affinity	AF7011
Two-Step Assay Kit (Goat Enhanced Polymer Assay System)	ZSGB-BIO	PV-9003
Universal two-step assay kit (mouse/rabbit enhanced polymer assay system)	ZSGB-BIO	PV-9000
